# Combined transnasal and transoral endoscopic approaches to the craniovertebral junction

**DOI:** 10.4103/0974-8237.65481

**Published:** 2010

**Authors:** Ivan H. El-Sayed, Jau-Ching Wu, Christopher P. Ames, Gopalakrishnan Balamurali, Praveen V. Mummaneni

**Affiliations:** Department of Otololaryngology-Head and Neck Surgery, UCSF Spine Center, University of California, San Francisco, San Francisco, USA; 2 Department of Neurosurgery, UCSF Spine Center, University of California, San Francisco, San Francisco, USA; 1 Department of Neurosurgery, Taipei Veterans General Hospital, National Yang-Ming University, Taipei, England; 3 Salford Royal Hospitals, Manchester, England

**Keywords:** Craniovertebral junction, endonasal, endoscopic, odontoidectomy, transnasal, transoral

## Abstract

**Objectives:**

To describe and evaluate a new technique of a combined endoscope-assisted transnasal and transoral approach to decompress the craniovertebral junction.

**Materials and Methods:**

A retrospective cohort of patients requiring an anterior decompression at the craniovertebral junction over a 12-month period was studied. Eleven patients were identified and included in the study. Eight of the patients had an endoscopic approach [endonasal (2), endooral (2), and combined (4)]. Four of the 8 patients in the endoscopic group had a prior open transoral procedure at other institutions. These 8 patients were compared with a contemporary group of 3 patients who had an open, transoral–transpalatal approach. Charts, radiographic images, and pathologic diagnosis were reviewed. We evaluated the following issues: airway obstruction, dysphagia, velopharyngeal insufficiency (VPI), length of hospital stay (LOS), adequate decompression, and the need for revision surgery.

**Results:**

Adequate anterior decompression was achieved in all the patients. The endoscopic cohort had a reduced LOS (*P* = 0.014), reduced need for prolonged intubation/tracheotomy (*P* =0.024) and a trend toward reduced VPI (*P* = 0.061) when compared with the open surgery group. None of the patients required a revision surgery.

**Conclusion:**

Proper choice of endoscopic transnasal, transoral, or combined approaches allows anterior decompression at the craniovertebral junction, while avoiding the need to split the palate. A combined transnasal–transoral approach appears to reduce procedure-related morbidity compared with open, transoral, and transpalatal surgeries.

## INTRODUCTION

Several surgical approaches provide anterior access to the craniocervical junction and the upper cervical spine, including transoral,[1,2] high transcervical,[3,4] and endoscopic transnasal–transoral approaches.[5‐11] The standard open transoral approach has gained wide acceptance by spine surgeons to treat ventral spinal cord compression at the C1–C2 level. However, to approach lesions of the craniovertebral junction, splitting of the palate is often required for adequate exposure. Mummaneni *et al* highlighted a surgical technique variation to avoid the palate split by using simple retraction of the soft palate with a red rubber catheter passed transnasally and secured to the uvula.[12] Despite this modification, in some circumstances, invasive approaches (ie, splitting the soft palate, resecting the hard palate, glossotomy, or midline mandibulotomy) are still required to provide surgical access to the craniovertebral junction. Such approaches are often used for decompression of lesions located high above the level of the palate. In addition, these invasive open approaches may also be needed in patients with atypical oral anatomy, or severe trismus (inability to distract the jaw open).

Palatal splitting has been reported to increase patient morbidity, especially velopharyngeal insufficiency (VPI), dysphonia, and dysphagia.[13] VPI occurs when there is incomplete closure of the nasopharynx with resultant escape of air and food into the nose during speech and swallow. Whereas dysphagia often resolves within 12 months following surgery, VPI often persists for a long term. We have recently referred 2 patients for pharyngoplasty after 1 year of persistent, significant VPI following an open transoral approach.

In order to avoid splitting the soft palate (and glossotomy/mandibulotomy, etc), we have used endoscopic transnasal–transoral techniques to decompress the craniovertebral junction in patients with challenging anatomic features.

## MATERIALS AND METHODS

A retrospective chart review was performed to review all the patients who underwent surgery of the craniovertebral junction during a 12-month period at our hospital (UCSF). We included only those with lesions located between the clivus and the body of C2. Eleven patients were identified and included. The medical records were reviewed for demographics and disease-specific information, including age, sex, diagnosis, surgical approach, length of hospital stay (LOS) after surgery, and surgery-related complications. The median age was 54 years (18–64 years). Eight of the patients had an endoscopic approach [endonasal (2), endooral (2), and combined endoscopic transnasal–transoral (4) approaches]. These 8 patients were compared with a contemporary group of 3 patients who had an open, transoral-transpalatal approach for lesions of the craniovertebral junction.

Diagnoses for the endoscopic group included infection (2), tumor (2), rheumatoid arthritis (1), and basilar impression (3). Four of the endoscopic patients had prior transoral surgery at another hospital in the past. All the 3 patients having an open approach had rheumatoid arthritis. None of these 3 had prior C1–C2 surgery.

Early and late postoperative complications were recorded, including documented VPI, dysphagia, need for insertion of percutaneous gastric feeding tube, and airway complications defined as need for endotracheal intubation longer than 24 h, or a tracheotomy as a result of the surgery. Preoperative and postoperative images [computed tomography (CT) and/or magnetic resonance imaging (MRI)] were carefully reviewed for evaluation of the adequacy of resection or decompression.

The data were stored in an excel spreadsheet and transferred to SAS (SAS Institute Inc., Cary, NC, USA) for data analysis by the UCSF Department of Biostatistics, using Fisher's exact test and the Mann-Whitney U test where appropriate.

### Surgical technique for endoscopic craniovertebral junction decompression

The patients were positioned supine and were intubated orally and given general anesthesia. Neuromonitoring with somatosensory-evoked potentials was used throughout the procedure. Spinal traction was applied as needed to get the odontoid process into a more normal position. Flouroscopy and neuronavigation were used for surgical guidance.

The endoscopic transnasal approach (endonasal approach) consisted of a bilateral approach through the nostrils. In the expanded form, as described by Kassam *et al*,[5] a nasal septal flap was first elevated for closure and then a corridor was prepared with a maxillary antrostomy, ethmoidectomy, middle turbinate resection on the right, posterior septectomy, and a wide sphenoidectomy. However, not all patients required sphenoidectomy or middle turbinectomy, and dissection was tailored to the individual's anatomy. Depending on the location of the lesion, the sphenoid floor and clival bone were drilled to access the craniocervical junction pathology. A midline incision was made with an extended needlepoint cautery through the posterior nasopharygeal mucosa down to the preveterbral fascia. The prevertebral muscles were dissected vertically in the midline and elevated laterally off the spine, which allowed exposure of the anterior tubercle of the atlas. Decompression was then performed using a drill, currettes, and/or Kerrison Rongeur.

**Figure 1:**
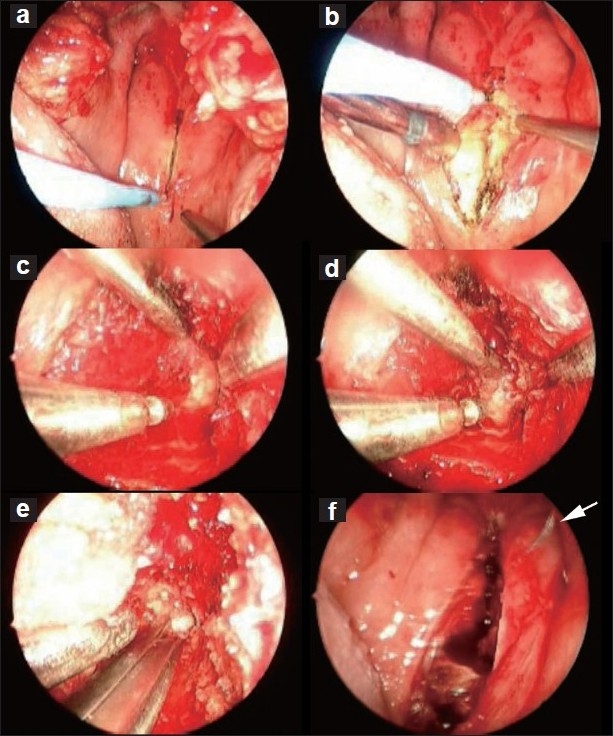
Intraoperative view of the anterior craniovertebral junction, using an endoscopic transoral approach. (a) Linear incision of the pharyngeal mucosa made by Bovie electrocautery. (b) Dissection and exposure of the underlying C1 anterior arch. (c) Drilling of the C1 anterior arch. (d) Drilling of the odontoid process. (e) Kerrison Rongeur used to remove the remnants of the odontoid and decompress the dura. (f) Closure of the pharyngeal wall following the decompression. (arrow indicates the suture needle)

The endosocopic transoral approach (endooral approach) was performed with soft palate retraction using 1 or 2 red rubber catheters tied to the uvula and pulled cranially through the nostrils.[12] The oral cavity and tongue were retracted open with a Spetzler-Sonntag oral retractor. The endoscope was guided under the retracted soft palate to visualize the posterior pharyngeal wall, and the pharyngeal incision was created and continued in the midline to the desired height to expose the C1–C2 area [Figure 1a, b]. The soft tissue and bony structures causing ventral cord compression were resected in a similar fashion to a transnasal decompression, described above [Figure 1c‐e]. The endoscope allowed us to "look" cranially above the level of the soft palate to complete the decompression.

For a combined transnasal and transoral approach, the exposure was a combination of the above-mentioned steps in both routes. Then the endoscope and surgical instruments were brought into the surgical field alternatively through the nose and mouth, in order to maximize the exposure with less dissection. Decompression was straightforward because visualization was gained from 2 different angles (from above and below the palate). The most favorable feature gained via combined transnasal and transoral approach is the ability to visualize laterally beyond the confines of the nasal cavity. Such lateral visualization is restricted by the nasal cavity/pterygoid plates in the transnasal-only approach. The addition of the transoral endoscopic approach increased the ability to reach out laterally beyond the confines of the transnasal approach.

CT-based image guidance navigation was typically used for the endoscopic cases. After the pharyngeal incision was completed, surgical dissection was performed with 2 surgeons working in tandem (otolaryngologist and neurosurgeon), one holding the endoscope and retraction while the other performed the dissection and decompression. Visualization of the pulsating dural sac and intraoperative fluoroscopic imaging of instruments placed at the borders of decompression confirmed the extent of the resection.

Closure of the pharyngotomy was performed with absorbable sutures [Figure 1f]. Tissue sealant and a transnasal merocel sponge were packed in the nose. A transoral feeding tube was then passed under endoscopic guidance. Postoperative CT scans or MRI were performed and adequate decompression was assured in every patient. If the clivus was resected as part of the dissection, a pedicled nasal septal flap was harvested and rotated over the clival defect for closure. This was held in place with an absorbable tissue sealant (DuraSeal, Covidien, Mansfield, MA, USA) and 2 transnasal merocel sponges.

## RESULTS

The outcomes of the 3 patients who underwent standard open transoral/transpalatal decompression of the craniovertebral junction were compared with those of the 8 patients who had endoscope-assisted decompression of the craniovertebral junction to evaluate the differences in the techniques. Issues such as postoperative airway obstruction, LOS, development of VPI, dysphagia requiring a nasogastric tube for more than 7 days, or the need for a percutaneous gastric feeding tube are detailed in Table 1. The hospital LOS was significantly reduced for patients undergoing the purely endoscopic approach to the craniovertebral junction compared with the open approach (*P* = 0.014). Furthermore, patients undergoing an open transoral approach had a statistically significant higher incidence of airway obstruction and tracheotomy (*P* = 0.024).

In the endoscopic group, 4 of the 8 patients had an open transoral approach in the past. When these 4 patients were grouped with those having an open approach currently and compared with the patients who had only an endoscopic approach, there was a trend toward reduction of VPI and dysphagia (*P* = 0.061) in patients who underwent a virgin endoscopic approach.[Table 1, right side]

**Table 1:** Summary of results

	Approach of craniovertebral surgery			Ever palate split		
	Endo (n = 8)	Open (n = 3)	*P*	Yes	No (n = 4)	*P*
LOS	7 (7–11)	15 (12–20)	0.014[1]			
Airway	1/8	3/3	0.024[2]	4/7	0/4	0.19[2]
VPI	2/8	3/3	0.06[2]	5/7	0/4	0.061[2]
Dysphagia > 7 days	2/8	3/3	0.061[2]	5/7	0/4	0.061[2]
Airway	1/8	3/3	0.024[2]	4/7	0/4	0.19[2]
PEG	0/8	1/3	0.061[2]	1/7	0/4	1.0[2]

The left side of the table demonstrates our experience with patients undergoing surgery for craniovertebral junction decompression with either a purely endoscopic approach or an open approach. Patients undergoing endoscopic procedures had a statistically lower rate of airway complications and a lower length of stay. The right side of the table ("Ever Palate Split") is shown since 4 patients in the endoscopic group had prior open surgery with palatal splitting. In this portion of the table, patients undergoing anterior decompression of the craniocervical junction with a history of prior palatal splitting were grouped with patients having a virgin open approach and compared with patients who had a virgin endoscopic-only approach. These data demonstrate that VPI and dysphagia tended to be lower in the virgin endoscopic surgery patients (*P* = 0.061). LOS = length of hospital stay (in days). Airway = patients requiring intubation for more than 24 h after surgery or requiring a tracheotomy as a result of the surgery.VPI = development of new onset velopharyngeal insuffi ciency occurring or lasting more than 2 months after surgery. Dysphagia = patients requiring supplemental feeding for more than 7 days after surgery. PEG = patients who required a percutaneous feeding tube after surgery. (1 = Mann-Whitney U test, 2 = Fisher's exact test).

## DISCUSSION

This study presents our early experience with a combined endonasal–endooral approach. While the approaches reported earlier include purely endonasal [5,6] or endoscopic transcervical approach,[3] our approach uses a flexible strategy with an endonasal approach or an endooral approach or a combined endonasal–endooral approach.[12] The combination of an endoscopic transnasal and transoral route appears to be a pragmatic way to conserve the advantages of endoscopic visualization via different corridors, while minimizing procedure-related morbidity due to splitting of the soft palate. We found that the endooral approach was advantageous in providing access to lesions that extended too far inferiorly to be reached by a purely endonasal approach.[6]

Moreover, in standard open transoral approaches with microscope visualization, the hard palate sometimes still obstructs visualization of the upper extent of the compressive lesion. The use of the endoscope overcame this obstacle with ease as it could be navigated to look around the palate.

Previously reported endoscopic transnasal odontoidectomy reports mentioned the most caudal limiting extent of the transnasal route to be the C1 rim (due to the position of the hard palate and the size of the nostril).[6] By combining the endonasal approach with a transoral endoscopic approach, we overcame this limitation and were able to reach lesions that extended into the mid-body of C2.

**Figure 2:**
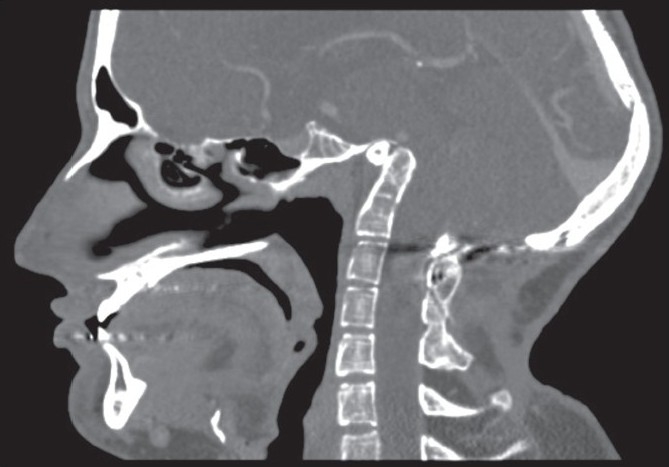
This patient had ventral brainstem compression at the tip of the odontoid. Note the extremely high location of odontoid, significantly above the palate in this patient with congenital platybasia. We used an endonasal approach alone to decompress this lesion

**Figure 3:**
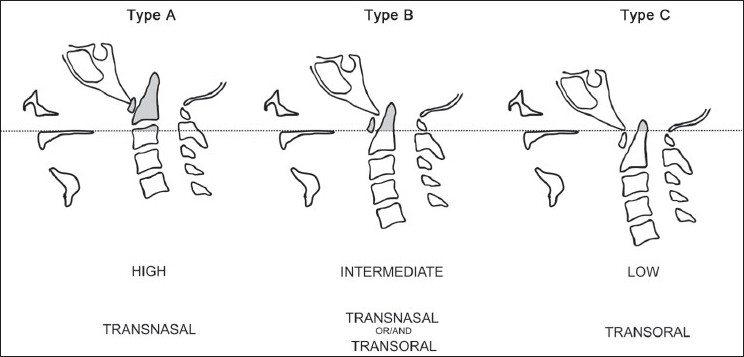
Schematic illustration of our algorithm to select the optimal choice of surgical approach. The relative position of the lesion to a line drawn from the hard palate to the posterior pharynx (the nasopalatal line) dictates the choice of approach. Lesions are defined as types A (well above NP line), B (intermediate location above the NP line), or C (at or below NP line). Left (Type A): For lesions located well above the hard palate, an endoscopic transnasal approach is optimal. Middle (Type B): For intermediately located compressive lesions of the craniovertebral junction that protrude above the hard palate, either a transnasal or a transoral endoscopic route may be used. Also, we found that a combination of both approaches was often quite helpful. Right (Type C): For lesions located at the level of the hard palate (or below) a standard open, transoral approach is preferred

Appropriate utilization of the combined transnasal and transoral endoscopic approach allowed for a minimally invasive surgery with full exposure for anterior decompression at the craniovertebral junction while avoiding a split of the soft palate. The optimal choice of a transnasal, transoral, or combined endoscopic approach should be tailored according to each individual's anatomy. Physical examination of the patient will reveal anatomic factors, such as trismus that would prevent oral exposure of the pharynx. We have also found that a careful review of the preoperative CT or MRI scan to evaluate the relative location of the hard palate and the target for decompression allows us to pick the optimal choice for a surgical approach. A radiographic line drawn along the floor of the palate to the posterior pharynx (the nasopalatal line) serves as an excellent reference point to assess the lesion location.[14] The lesions can be categorized as types A (high above the nasopalatal NP line= nasopalatal line [Figure 2], B (intermediate location above the NP line), and C (at the level of (or below) the NP line). For intermediately located lesions (Type B), either an endoscopic transnasal or an endoscopic transoral approach may be adopted for decompression. Such lesions may also be easily accessed using a standard, open transoral approach without a palate split as reported by several authors.[1,2,12,15‐17] Finally, for low lying lesions or lesions extending to the midbody of C2 (Type C, at or below the NP line), an endoscopic transoral approach may be used [Figure 3].

We found that the endoscopic approach significantly reduced the LOS and postoperative airway compromise when compared with a standard transoral/transpalatal open approach. The increased airway obstruction and LOS in the open transoral group was probably related to the increased oropharyngeal edema and muscular dysfunction caused by the incision of the palate and posterior pharynx as well as by prolonged retraction on the tongue.

Limitations to the endoscopic approach include 2-dimensional visualization, a relatively small working space, and the learning curve associated with endoscopic technology. Consequently, open transoral surgery continues to be our primary choice of approach in cases that have an intermediate position of the odontoid (not blocked by the position of the hard palate).

There are several drawbacks to our study. First, variables in the patient's disease and anatomy were not controlled in this retrospective analysis. The open procedures were done during the earlier phase of the study time period, whereas the endoscopic procedures were done during the later phase of the study. The data represented our early experience with the endoscopic procedure (for which there is a learning curve). The other issue was that this series was based on our referral patterns at a major tertiary care center, which might be biased toward more complicated patients. This was evidenced by the fact that 4 of the 8 patients undergoing the purely endoscopic procedure had a prior open transoral procedure in the past at an outside hospital. Although revision surgeries might lead to a higher complication rate, our ability to perform adequate resections endoscopically after a prior open procedure further demonstrates the feasibility of the endoscopic approach.

## CONCLUSION

The combined endonasal–endooral approach is a useful approach offering a wide access to the anterior craniovertebral junction. This approach appears to reduce airway obstruction and LOS after surgery when compared with a standard open transoral/transpalatal approach.
